# Topological integration of RPPA proteomic data with multi-omics data for survival prediction in breast cancer via pathway activity inference

**DOI:** 10.1186/s12920-019-0511-x

**Published:** 2019-07-11

**Authors:** Tae Rim Kim, Hyun-Hwan Jeong, Kyung-Ah Sohn

**Affiliations:** 1 Department of Computer Engineering, Ajou University, Suwon, 16499 South Korea; 2 Department of Molecular and Human Genetics, Baylor College of Medicine, Houston, TX 77030 USA; 3 Jan and Dan Duncan Neurological Research Institute, Texas Children’s Hospital, Houston, TX 77030 USA

**Keywords:** Multi-omics data, Integrative analysis, Random walk, Reverse phase protein Array, Pathway-based analysis, Network propagation, Breast cancer, Survival prediction

## Abstract

**Background:**

The analysis of integrated multi-omics data enables the identification of disease-related biomarkers that cannot be identified from a single omics profile. Although protein-level data reflects the cellular status of cancer tissue more directly than gene-level data, past studies have mainly focused on multi-omics integration using gene-level data as opposed to protein-level data. However, the use of protein-level data (such as mass spectrometry) in multi-omics integration has some limitations. For example, the correlation between the characteristics of gene-level data (such as mRNA) and protein-level data is weak, and it is difficult to detect low-abundance signaling proteins that are used to target cancer. The reverse phase protein array (RPPA) is a highly sensitive antibody-based quantification method for signaling proteins. However, the number of protein features in RPPA data is extremely low compared to the number of gene features in gene-level data. In this study, we present a new method for integrating RPPA profiles with RNA-Seq and DNA methylation profiles for survival prediction based on the integrative directed random walk (iDRW) framework proposed in our previous study. In the iDRW framework, each omics profile is merged into a single pathway profile that reflects the topological information of the pathway. In order to address the sparsity of RPPA profiles, we employ the random walk with restart (RWR) approach on the pathway network.

**Results:**

Our model was validated using survival prediction analysis for a breast cancer dataset from The Cancer Genome Atlas. Our proposed model exhibited improved performance compared with other methods that utilize pathway information and also out-performed models that did not include the RPPA data utilized in our study. The risk pathways identified for breast cancer in this study were closely related to well-known breast cancer risk pathways.

**Conclusions:**

Our results indicated that RPPA data is useful for survival prediction for breast cancer patients under our framework. We also observed that iDRW effectively integrates RNA-Seq, DNA methylation, and RPPA profiles, while variation in the composition of the omics data can affect both prediction performance and risk pathway identification. These results suggest that omics data composition is a critical parameter for iDRW.

**Electronic supplementary material:**

The online version of this article (10.1186/s12920-019-0511-x) contains supplementary material, which is available to authorized users.

## Background

Advances in high-throughput sequencing technologies and their integration, including genome, transcriptome, epigenome, and proteome sequencing, has shifted perspectives from the micro-level to the macro-level in biological research. Certain phenomena can be observed in each omics layer, enabling researchers to understand the complex interactions within and between biological mechanisms. Several studies have proposed methods for integrating omics data obtained from high-throughput sequencing in different layers to provide insights into systems biology [1–4]. In cancer research, integrative models for multi-omics data not only greatly improve clinical prognosis predictions but also allow cancer-related biomarkers to be identified [5–12]. For example, Kim et al. proposed a graph-based framework for integrating multi-omics data (including CNA, DNA methylation, miRNA, and gene expression data) with prior knowledge to improve the clinical outcome prediction performance for glioblastoma multiformes and serous cystadenocarcinoma [13]. In addition, Bertrand et al. developed a patient-specific data integration framework (OncoIMPACT) to identify driver genes using the scored impact of single nucleotide polymorphism (SNP), indel, and copy number variation (CNV) data for individual patients with one of five different cancer types [14]. Their prediction of patient-specific driver genes using OncoIMPACT was validated with in silico and in vitro experiments, with their model proving to be more robust and precise than the baseline model.

Although many previous studies have focused on the integration of multi-omics data, proteomic data is rarely used for integrative multi-omics data analysis. Proteins are a fundamental unit of a biological complex, in which they have a functional role, and protein expression data can be utilized for the diagnostic prognosis of cancer patients [15–18]. However, combining protein-level and gene-level data is challenging because the relationship between the two data types is unclear [19]. Not all genomic variation is translated into proteins via perturbations (e.g. post-transcriptional regulation or post-translational regulation and modification) [20, 21], thus gene- expression data may not accurately reflect active cellular function [22, 23]. Furthermore, other limitations, such as mass spectrometry-based technology for the quantitative analysis of proteins, which exhibits low sensitivity for low-abundance proteins, make it difficult to collect information on cancer-related signaling proteins [24].

The reverse phase protein array (RPPA) is an antibody-based protein assay platform for high-throughput sequencing that quantifies the expression of a target protein. It is cost-effective and highly sensitive to the target protein, even at low concentrations [24]. However, it greatly relies on the quality of the antibodies and requires screening to select the appropriate antibodies for the corresponding target proteins. Although RPPAs face these limitations, several studies have adopted the RPPA platform to produce proteomic data for the detection of cancer tissue phenotypes. Additionally, The Cancer Genome Atlas (TCGA) has also generated and made public RPPA data for TCGA cancer samples for use in characterizing various cancer types. This data is publicly accessible in The Cancer Proteomics Atlas (TCPA) [25].

Pathway-level analysis is necessary because it is not possible to define biological functions via a single gene or a single molecule. The Kyoto Encyclopedia of Genes and Genomes (KEGG) [26] provides pathway information that represents a functional network consisting of a set of gene products (such as proteins and functional RNA) and their relationship. Based on this pathway information, several previous studies have utilized gene- or protein-level expression data to infer pathway activity [27–29]. These studies have shown that pathway-level information provides a better description of disease phenotypes than does gene-level information. Furthermore, previous research employing directed random walk on a gene-metabolite graph (DRW-GM) [30] and integrative directed random walk (iDRW) [31] has proposed a method for the integration of multi-omics into pathway information based on [27]. DRW-GM involves the pathway-based integration of gene expression and metabolite data, while iDRW, which was proposed in our previous study, utilizes the pathway-based integration of RNA-Seq and DNA methylation profiles. Both methods demonstrate improved performance and reveal that the integration of multi-omics data into pathway information is useful for predicting disease phenotypes and identifying risk pathways.

In this study, we propose a pathway-based integration method for RPPAs and other omics data. Specifically, we focus on the utilization of RPPA data in which the proteins are sparse with respect to the pathway gene set. In our experiments, we merge RNA-Seq, DNA methylation, and RPPA profiles into a single pathway profile that is employed for survival prediction and risk-pathway identification in breast cancer.

## Methods

### Data

**Fig. 1:**
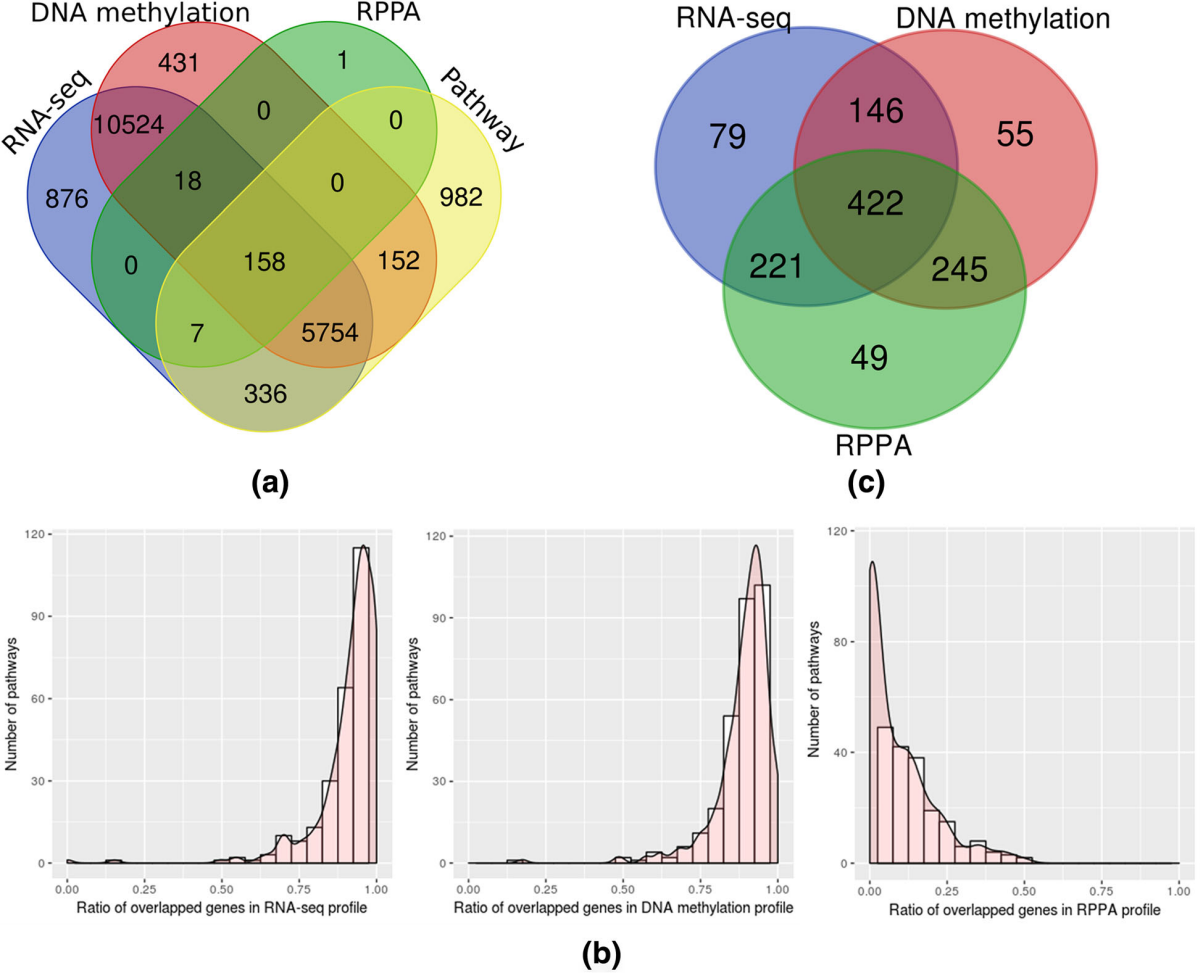
Distribution of RNA-Seq, DNA methylation, RPPA profile, and KEGG pathway data. (**a**). Venn diagram for genes (or proteins) in RNA-Seq, DNA methylation, RPPA profile, and KEGG pathway. Venn diagram for showing the distribution of logical relation among genes (or proteins) in each profile (Venn diagram was drawn using a tool in this website - http://bioinformatics.psb.ugent.be/webtools/Venn/). (**b**). Distribution of the ratio of overlapping genes (or proteins) with those genes in each pathway. The frequency over the ratio of overlapping genes (or proteins) in each omics profile with genes in each pathway is shown as histogram and density plot. (**c**). Venn diagram for the number of samples in each omics profile

We downloaded pathway annotation files from KEGG [32]. Of these pathways, we selected 327 human pathways containing 7389 gene features. Level 3 multi-omics profiles for breast cancer were collected from the TCGA breast cancer dataset from the Broad Institute GDAC Firehose [33]. We employed RNA-Seq, DNA methylation, and RPPA profiles as transcriptomic, epigenomic, and proteomic data, respectively. The RNA-Seq profile contained 17,673 genes from 869 samples, the DNA methylation profile 17,037 genes from 868 samples, and the RPPA profile 188 proteins from 937 samples. For missing values, we imputed the median of the corresponding gene, protein, or methylation. We analyzed the common features of the pathways and each omics profile, as shown in Fig. 1a. In order to assess the level of sparsity in the RPPA profile, we calculated the ratio of matched genes in each omics profile with the genes in each pathway. As shown in Fig. 1b, the RNA-Seq and DNA methylation profiles included almost a full set of genes for each pathway, while the RPPA profile contained only a few matched proteins for each pathway. The 422 samples found in common in all three profiles were extracted (Fig. 1c). They contained clinical information on vital status and survival period. We excluded samples for which the survival period was missing or negative. Those samples whose vital status was reported as 1 (living) but for whom the survival period was less than three years were also excluded, leaving a total of 376 extracted samples. For survival analysis, we classified each patient into one of two groups: patients whose survival exceeded three years were placed in the long-term survival group, while those with a survival period of less than three years were placed in the short-term survival group [34]. Of the 376 extracted samples, 177 exhibited long-term survival (≥ 3 years) and 199 exhibited short-term survival (< 3 years).

### Overview of the proposed framework

**Fig. 2:**
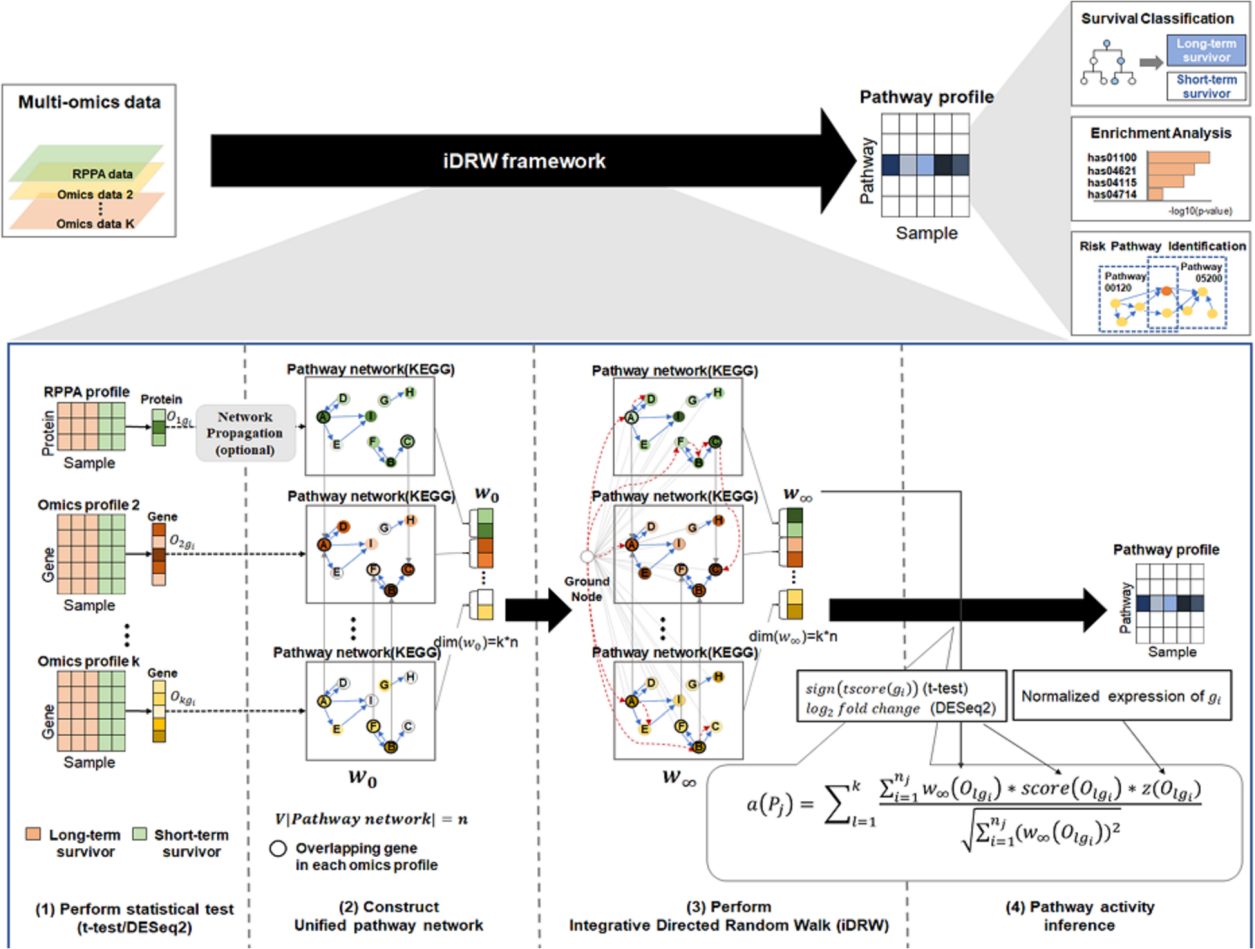
Overview of the proposed framework

In order to integrate the RPPA profile with other multi-omics data and merge them into a single pathway profile, we deployed and extended the iDRW framework [31]. The overall process of the proposed framework is illustrated in Fig. 2. Our proposed framework included four steps, which are explained briefly below.

1. Statistical testing (*t*-tests or DESeq2) was conducted to obtain initial gene weights that indicate the extent to which a gene (or protein) differentiates between long-term and short-term survival for breast cancer.
2. A unified pathway network was conducted for the iDRW method that reflected the topological information of the pathway. Each gene (or protein) weight vector was assigned to the corresponding pathway network. Given that the RPPA profile contains few protein expressions (approximately 180 proteins) and that there is only a very small overlap between the genes corresponding to proteins in the RPPA profile and the genes in the pathway, many of the initial weights for the RPPA profile were missing or assigned as zero. We conducted random walk with restart (RWR), which represents a network propagation algorithm, on the protein-level pathway network to estimate the initial weight of the missing proteins.
3. iDRW was utilized to integrate the initial weight of each gene (or protein) into the unified pathway network. In the iDRW approach, the initial gene (or protein) weights are mixed to reflect the topological information of the pathway. Thus, we obtained a final weight vector that represented the topological importance of each gene in terms of distinguishing between the long-term and short-term survival groups.
4. A pathway profile using the pathway activity inference method derived from [27] was constructed. Given the pathway profile, we performed survival prediction using a random forest classifier and identified risk pathways for breast cancer. Additionally, in this step, we investigated the contribution of different combinations of each omics type.

A more detailed description of these steps is given in the following sections.

### Unified pathway network construction

The iDRW framework operates on a unified pathway network that consists of a set of pathway networks corresponding to the number of omics types. We first constructed pathway networks for each of the RNA-Seq, DNA methylation, and RPPA profiles and combined them into a unified pathway network. A pathway network for a single omics profile contains relational information for biological molecules (such as genes, gene products, or chemical compounds) from the KEGG database [32]. We used genes or gene products as nodes for the pathway network and their relationships as edges. We used the KEGGgraph R package, which facilitates the conversion of KEGG pathway information into network objects [35]. For the 327 pathways that had the pathway id prefix *hsa*, a pathway network was generated containing a total of 7389 nodes and 58,399 directed edges. In this paper, we refer to the generated pathway networks as the transcriptome network, epigenome network, and proteome network for the RNA-Seq, DNA methylation, and RPPA profiles, respectively.

**Fig. 3:**
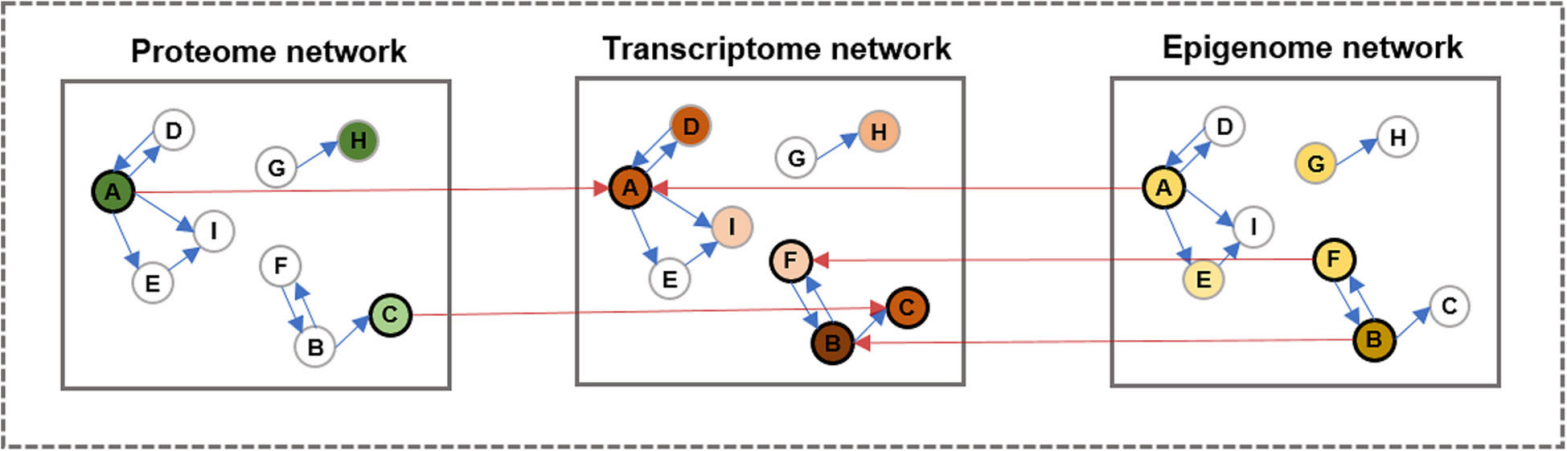
Structure of the unified pathway network. A colored node indicates that the gene is included in the corresponding omics profile, and a white node indicates that it is included in a pathway gene set although not in omics profile. A node with a bold borderline represents that the gene appears in both corresponded profile and RNA-Seq profile. In this structure, the inter-relation edges are assigned between the nodes containing the same gene feature in RNA-Seq profile, and the edge direction is set from a proteome network to a transcriptome network or from an epigenome network to a transcriptome network

To generate the unified pathway network, it was necessary to connect the three pathway networks. Figure 3 presents the structure of the unified pathway network. It should be noted that the structure was designed to obtain better-integrated gene or protein scores rather than reflecting biological relationships. A detailed description on the same is given in the following sections. As shown in Fig. 3, each node represents a gene in a pathway network. The inter-relationship edges are assigned to genes that overlap two different omics profiles on the corresponding pathway networks. For example, the RNA-Seq profile contains the gene *AKT1*, while the RPPA profile contains an antibody corresponding to the AKT1 protein; thus, a directional edge is assigned to the node for AKT1 in the transcriptome network and the node for AKT1 in the proteome network. In this structure, we assign directional edges to the overlapping genes (with the inter-network edge following the direction of epigenome network → transcriptome network and proteome network → transcriptome network). We selected this structure for the unified pathway network empirically based on the best performance in survival classification for breast cancer (See Additional file 1: Supplementary Material 1 for more information about the network structures used in this experiment).

### Topological integration of multi-omics profiles based on iDRW

To apply the iDRW method to the unified pathway network, an initial weight vector was assigned to the network. The initial vector *W*_0_ for each omics profile was defined as follows:

$$ {W}_0=-\mathit{\log}\left({w}_g+\varepsilon \right) $$

where *w*_*g*_ denotes the weight of the gene *g* in the unified pathway network and *ϵ* = 2.2 ∗ 10^−16^. Additionally, *w*_*g*_denotes the min-max normalized *p*-value of gene *g* obtained using a different statistical method from an omics profile. We performed a two-sided *t*-test for the DNA methylation and RPPA profiles, while the differential expression test DESeq2 [36] was employed for the RNA-Seq profile.

In the iDRW method, a random walker starts at the ground node and then moves onto a randomly selected neighbor node or returns to the ground node with restart probability *r* at each time step *t*. The process is defined as follows:

$$ {W}_{t+1}=\left(1-r\right){M}^T{W}_t+{rW}_0 $$

where *W*_*t*_ denotes the weight vector that represents the probability of being at each node at time step *t*; *M* denotes a row-normalized adjacency matrix of the unified pathway network; *r* is the restart probability for the random walker; *W*_0_ is the initial weight vector; and *W*_*t*_ is updated at each time step and converges to steady-state *W*_∞_ when |*W*_*t* + 1_ − *W*_*t*_| ⋅  <  ⋅ 10^−10^ as guaranteed by [37]*.* We performed parameter tuning for *r* in the range of [0.2, 0.4, 0.6, 0.8].

### Estimation of missing values in the RPPA profile using a network propagation algorithm

Because the *W*_0_ of the RPPA profile is extremely sparse, we employed a network propagation algorithm to estimate the putative expression levels of the missing proteins in the pathway via the diffusr package (https://github.com/dirmeier/diffusr). With diffusr, RWR was used for missing value estimation. *r*_*i*_^*t*^ denotes the propagated weight vector at time step *t* at node *i*, and is obtained by RWR [38] as follows:

$$ {r}_i^{t+1}={pAr}_i^t+\left(1-p\right){e}_i $$

where *A* denotes the column-normalized adjacency matrix of a given network; *e*_*i*_ is the *W*_0_ for the RPPA profile at node *i*; and *p* is the restart probability, which controls how much of the local topological information is reflected when the random walk converges to its steady state. Thus, the higher the restart probability, the more local the topological information, and the lower the restart probability, the more global the information. *p* was tuned by a grid search on [0.2, 0.4, 0.6, 0.8]. At each time step, ${r}_i^{t+1}$ was updated, and the steady-state weight vector *r*_*i*_^∞^ was obtained from a fixed number of iterations. *r*_*i*_^∞^ was used as *W*_0_ for the RPPA profile, and the *W*_0_ for each omics profile was assigned to the corresponding pathway network.

### Pathway activity inference

Pathway activity inference using a gene profile was conducted using the method suggested by [27]. To infer the pathway activity using a multi-omics profile, we redefined the general inference method for the activity of the *j*-th pathway as

1 $$ \alpha \left({P}_j\right)={\sum}_{l=1}^k\frac{\sum_{i=1}^{n_j}{w}_{\infty}\left({O}_{\lg i}\right)\ast score\left({O}_{\lg i}\right)\ast z\left({O}_{\lg i}\right)}{\sqrt{\sum_{i=1}^{n_j}{\left({W}_{\infty}\left({O}_{\lg i}\right)\right)}^2}} $$

where *P*_*j*_, which corresponds to the *j*-th pathway, contains *n*_*j*_ differentially expressed genes $\left({g}_1,{g}_2,\dots, {g}_{n_j}\right)$ in which the *p*-value (*w*_*g*_) is < 0.05; *k* is the number of omics profiles employed; *O*_*l*_ represents the *l*-th omics profile used when inferring the pathway activity; ${O}_{l{g}_i}$ represents gene *g*_*i*_ in *O*_*l*_; ${W}_{\infty}\left({O}_{l{g}_i}\right)$ is the weight of *g*_*i*_ calculated in *O*_*l*_; and $z\left({O}_{l{g}_i}\right)$ is the normalized value for the expression of *g*_*i*_ in *O*_*l*_. In addition, in the RNA-Seq profile, which consists of count-based data, $score\left({O}_{l{g}_i}\right)$ is the *log*_2_*fold change* from the DESeq2 method [36] for *g*_*i*_ in *O*_*l*_. In the DNA methylation and RPPA profiles, $score\left({O}_{l{g}_i}\right)$ is the $\mathit{\operatorname{sign}}\left( tscore\left({O}_{l{g}_i}\right)\right)$ from a two-tailed *t*-test. This process generates the pathway activity profile *P*_*j*_, which is used as an input feature for survival classification.

To investigate the contribution of each omics type to survival prediction within the same model, we experimented with variants of the aforementioned pathway activity inference formula. *P* denotes a power set (excluding the null set) of *k* omics profiles {*O*_1_, *O*_2_ … *O*_*k*_}, which is used as the input profile. In this study, we used three omics profiles so that the power set of {*O*_1_, *O*_2_, *O*_3_} can be denoted as

$$ P=\left\{\left\{{O}_1\right\},\left\{{O}_2\right\},\left\{{O}_3\right\},\left\{{O}_1,{O}_2\right\},\left\{{O}_1,{O}_3\right\},\left\{{O}_2,{O}_3\right\},\left\{{O}_1,{O}_2,{O}_3\right\}\right\}. $$

We denote the *r*-th subset of *P* as *S*_*r*_. The refined pathway activity inference formula is as follows:

2 $$ \alpha \left({P}_j\right)={\sum}_{l=1}^k\frac{\sum_{i=1}^{n_j}{w}_{\infty}\left({S}_{r\mathrm{g}i}\right)\ast score\left({S}_{r\mathrm{g}i}\right)\ast z\left({S}_{r\mathrm{g}i}\right)}{\sqrt{\sum_{i=1}^{n_j}{\left({W}_{\infty}\left({S}_{r\mathrm{g}i}\right)\right)}^2}} $$

Eqs. 1 and 2 are almost identical except for the number of omics types used to infer the pathway activity. We used Eq. 2 to measure the predictive power of the combination of each omics type and determined the optimal combination for survival prediction. For example, the RNA-Seq, DNA methylation, and RPPA profiles were used as an input to produce *iDRW*(*GMP*) and the RNA-Seq and DNA methylation profiles were used to produce *iDRW*(*GM*). When generating the pathway profile in *iDRW*(*GMP*) using RNA-Seq, DNA methylation, and RPPA data to infer the pathway activity score, *O*_*l*_ contains {RNA-seq, DNA methylation, RPPA} and *k* = 3 for Eq. 2. However, when RNA-Seq and RPPA data was used in *iDRW*(*GMP*) to infer pathway activity, *O*_*l*_ contained {RNA-seq, RPPA} and *k* = 2 for Eqs. 1 and 2 was used for cases in which different omics combinations were used as input to calculate the pathway activity score.

### Survival classification and evaluation

In our study, we conducted survival analysis using a binary classification of long-term survival (surviving more than three years) and short-term survival (surviving fewer than three years) using the pathway profiles. In our dataset, 177 samples exhibited long-term survival and 199 exhibited short-term survival. Using the pathway activity profile, we first extracted the top-*N* pathways from among the 327 pathways that exhibited the best performance for survival classification to obtain an optimal pathway list that significantly differentiated between the long-term and short-term survival groups. To achieve this, we sorted all pathways by increasing *p*-value from the two-tailed *t*-tests for the pathway activity (i.e., pathways with a lower *p*-value were ranked higher). Based on this ranking, we selected the top *k* pathways, and the model was then evaluated using 5-fold cross validation (with the caret R package [39]) via a random forest classifier (with therandomForest [40] R package) and varying *k* = 5, 10, …, for half of the total pathways. The procedure was repeated ten times for reliability. With the top-*N* pathways chosen, we performed leave-one-out cross-validation (LOOCV) using the caret R package [39] in a dataset with *n* observations via a random forest classifier for survival classification. Random forest is composed of several decision trees. A decision tree makes decision rules that enable a correct decision for the target label. A random forest classifier can be used for non-linear datasets and is also robust to overfitting [41].

## Results and discussion

### Integrative analysis utilizing the RPPA profile to achieve accurate survival prediction

**Fig. 4:**
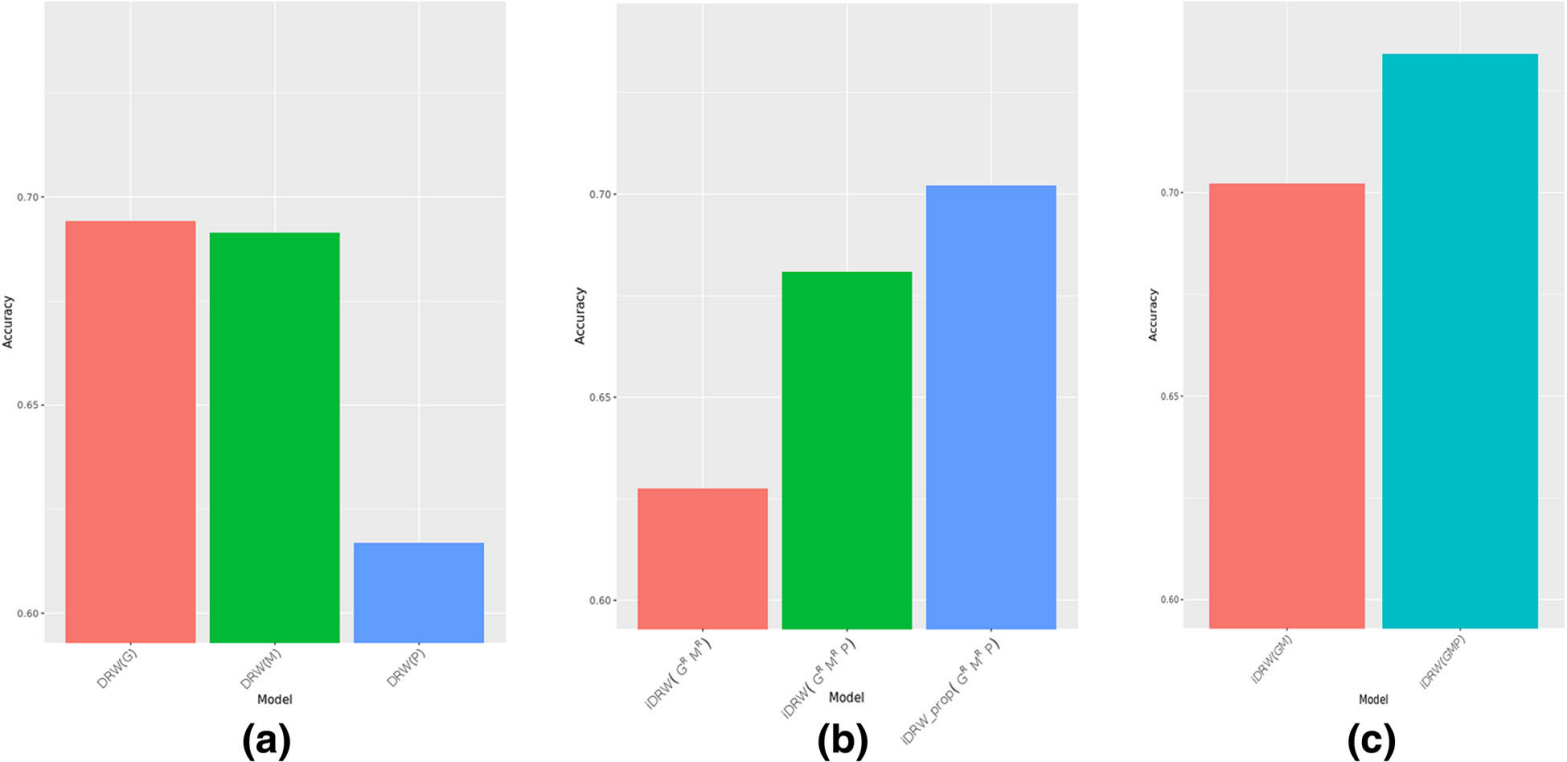
Performance comparison between different methods and profiles. (**a**). In case of using a single omics profile. *DRW*(*G*) used RNA-Seq profile; *DRW*(*M*) used DNA methylation profile; *DRW*(*P*) used RPPA profile. (**b**). In case of using reduced RNA-Seq and DNA methylation profile. Each profile was reduced to include genes overlapping with RPPA proteins. *iDRW*(*G*^*R*^*M*^*R*^) used reduced RNA-Seq and reduced DNA methylation profile; *iDRW*(*G*^*R*^*M*^*R*^*P*) used reduced RNA-Seq, reduced DNA methylation, and RPPA profile; *iDRW*_*prop*_(*G*^*R*^*M*^*R*^*P*) performed network propagation using RWR on the proteome network. (**c**). Performance comparison of *iDRW*(*GM*) and *iDRW*(*GMP*). *iDRW*(*GM*) is a previous method which used RNA-seq and DNA methylation profile. *iDRW*(*GMP*) is our proposed model which used RNA-seq, DNA methylation, and RPPA profile in this study

The pathway-based prediction model created using the RPPA profile exhibited significantly lower accuracy than that created using the RNA-Seq profile or the DNA methylation profile (Fig. 4a). We suspect that this is due to the sparsity of RPPA proteins. To verify this, we developed an alternative experimental setting by filtering the RNA-Seq and DNA methylation profiles to include only genes that overlapped with the corresponding RPPA proteins. As a result, the RNA-Seq profile included 183 genes and the DNA methylation profile included 176 genes (Fig. 1a). *iDRW*(*G*^*R*^*M*^*R*^) denotes the pathway-based integration model obtained using the reduced RNA-Seq and DNA methylation profiles for survival classification. The addition of the RPPA profile to this model is denoted as *iDRW*(*G*^*R*^*M*^*R*^*P*). As shown in Fig. 4b, the addition of the RPPA profile improved the survival classification performance. The *iDRW*_*prop*_(*G*^*R*^*M*^*R*^*P*) model, which utilized the propagated proteome network, produced an accuracy exceeding that of the *iDRW*(*G*^*R*^*M*^*R*^*P*) model.

We also compared the performance of *iDRW*(*GMP*) with *iDRW*(*GM*) that is our previously proposed model (Fig. 4c). Compared with *iDRW*(*GM*), *iDRW*(*GMP*) had a greater survival prediction accuracy. This indicates that, although the RPPA profile does not exhibit a high accuracy when used alone, it can be used to effectively discriminate between the long-term and short-term survival groups when employed with other omics profiles to create a pathway-based prediction model. To illustrate the overall prediction performance for the survival period, we generated two additional survival curves using the survival [42] R package for *iDRW* (*GM*) and *iDRW*(*GMP*) (see Additional file 2: Supplementary Material 2a and b). The difference between the survival curves for the long-term and short-term group was statistically significant based on chi-square tests for both models (*p* = 2e-07 and *p* = 5e-13, respectively). Based on the much lower *p*-value for *iDRW*(*GMP*), it is clear that the RPPA data significantly improves survival classification performance for TCGA breast cancer data. In addition, it can be concluded that network propagation using the proteome network is a reasonable approach for addressing the sparsity of the RPPA profile.

### Predictive power of different omics type combinations when inferring pathway activity scores

**Fig. 5:**
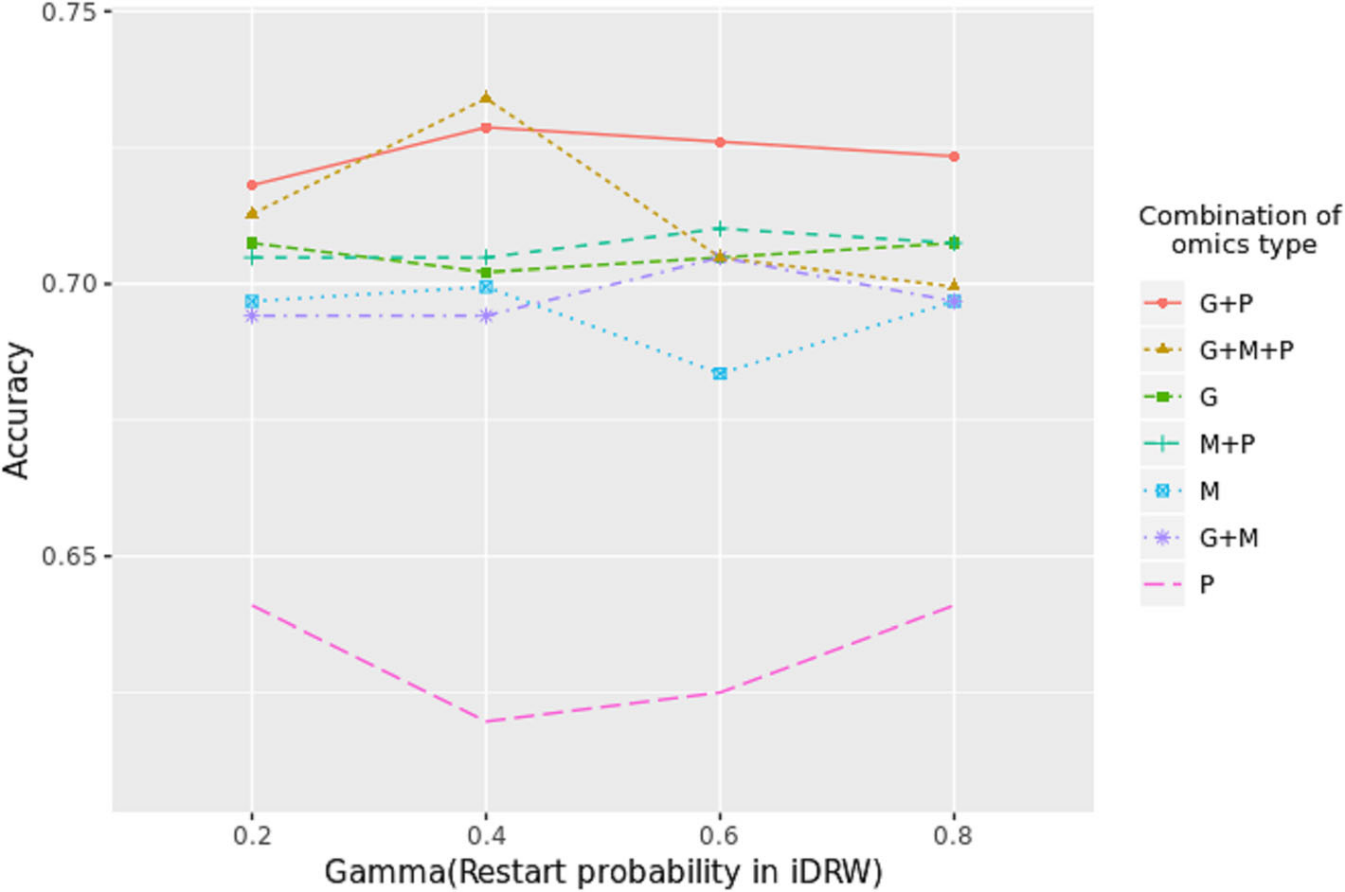
Classification accuracy using different combination of each omics type in pathway activity score calculation. Each case in legend means the combination of omics profiles which was used to calculate the pathway activity score. All cases in this experiment were originating from *iDRW*(*GMP*) model (status before pathway activity inference step) with varying γ

To investigate the effect of different combinations of omics profiles on survival prediction, we conducted pathway activity inference with different combinations of omics profiles. As shown in Fig. 2, the experiment was performed using *iDRW*(*GMP*) until Step (3), and Eq. 2 was used on different combinations only in Step (4). Prediction accuracy was compared by varying γ (Fig. 5). Interestingly, the (*G + P*) combination, which used information from the RNA-Seq and RPPA profiles in pathway activity inference, exhibited superior performance in survival prediction, while (*P*), which used information from the RPPA profile only, exhibited the worst performance. (*G + M + P*), which used information from all of the three profiles to infer pathway activity, had the best accuracy when γ = 0.4. However, (*G + P*) generally exhibited a more stable and higher overall accuracy and was robust with respect to γ.

### Performance comparison of pathway-based integration models for survival classification

**Fig. 6:**
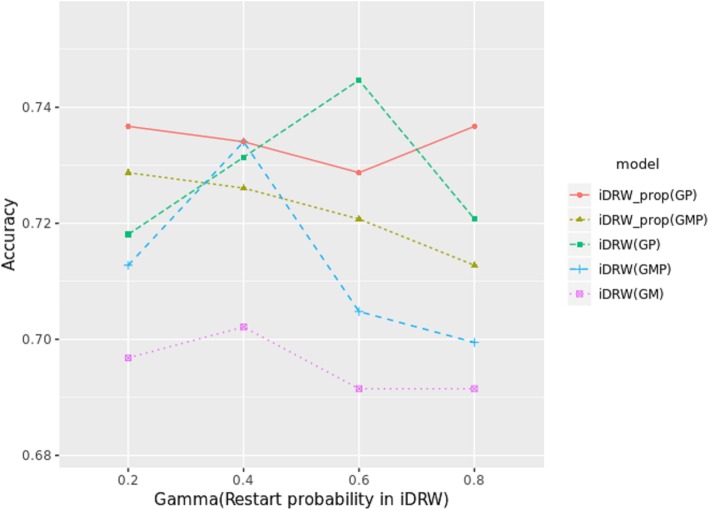
Performance comparison of pathway-based integration model with varying γ

**Fig. 7:**
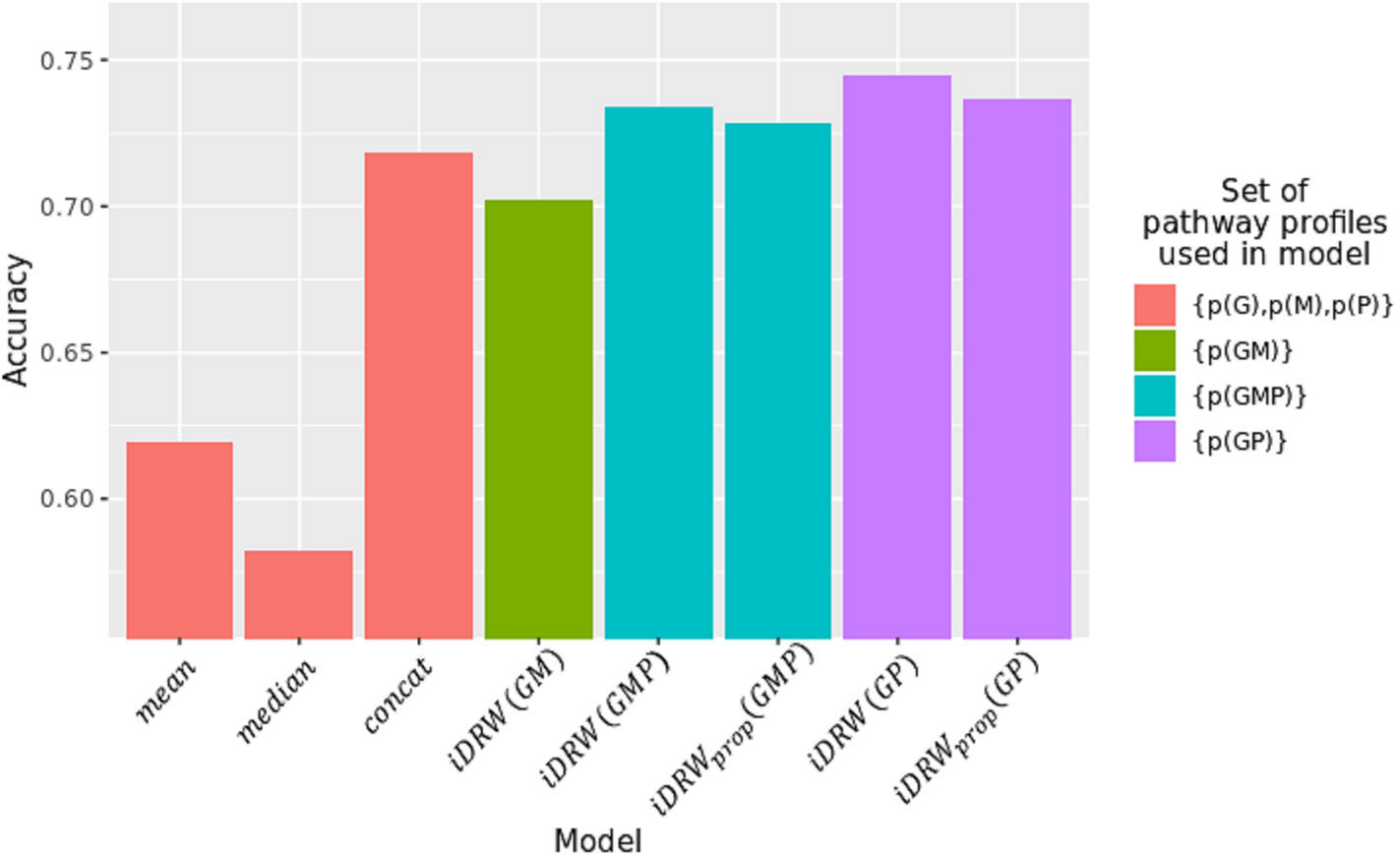
Performance comparison of the pathway-based integration model with optimized γ

Based on the aforementioned results, we compared each pathway-based integration model by varying the restart probability γ for the iDRW method (Fig. 6). Unlike the aforementioned experiments, the omics profiles used as inputs and the omics profiles used to infer the pathway activity were identical. As shown in Fig. 6, *iDRW*(*GP*) produced the best performance for survival classification when γ = 0.6. However, *iDRW*_*prop*_(*GP*) exhibited both stable and higher performance. For the *iDRW*_*prop*_ model, the optimized restart probability for proteome network propagation was used for each γ in [0.2, 0.4, 0.6, 0.8] to compare the performance of all models with respect to γ (Fig. 6). All models using the RPPA profile (*P*) exhibited better performance when compared with the *iDRW*(*GM*) model presented in our previous study. Interestingly, the *iDRW*_*prop*_ model had a slightly lower performance (though it was more stable) with changes in γ than that of the *iDRW* model. This indicates that the *iDRW*_*prop*_ model is more robust to changes in the restart probability than is *iDRW*, and that network propagation mitigates the sparsity problem in RPPA data. Based on the optimized models obtained from the experiment presented in Fig. 6, we measured the final performance of all models for survival classification. As baseline models, we considered the mean, median, and concat models. The mean and median models computed either the mean or median of the normalized expression values of the pathway gene members in order to construct a pathway profile. The concat model was constructed using the simple concatenation of the pathway profiles obtained from the RNA-Seq, DNA methylation, and RPPA profiles in order to demonstrate the utility of our iDRW framework. The others were generated using an iDRW-based method that enables the integration of each omics profile in a pathway profile on the unified pathway network. As can be observed in Fig. 7, *iDRW*(*GP*) exhibited the best performance for survival classification. All models created using the RPPA profile out-performed models without the RPPA profile. As a result, it is clear that RPPA data is useful for the prediction of long-term or short-term patient survival.

### Identification of risk pathways and systemic analysis of iDRW(GP) and iDRW(GMP)

We identified the top 10 risk pathways from the ranked pathway list for *iDRW*(*GP*) and *iDRW*(*GMP*). The ranked pathway list was obtained from the *p*-values for two-tailed *t*-tests of the pathway activity with respect to long-term and short-term survival. The importance score was calculated based on prediction error using the out-of-bag (OOB) estimate method. OOB estimation is the mean prediction error of random forest without training sample *x*_*i*_. The importance of a feature was defined by the degree to which the prediction error increased when *x*_*i*_ was excluded. The score was scaled from 0 to 100 with the caret R package [39].

Table 1 shows the top 10 risk pathways for *iDRW*(*GP*). It can be observed that the list contains several pathways that have been previously reported to have a direct association with cancer. The *p53 signaling pathwa*y is a well-known anti-cancer pathway that plays a role in apoptosis and as a tumor suppressor [43–45]. The pathway of *pathways in cancer* was also extracted [46]. Cellular senescence ceases cell division and suppresses premalignant cell proliferation [47]. It also exhibits age-related pathology and its abnormal function promotes cancer progression [47–50]. The metabolic pathway, which was the top-ranked risk pathway for *iDRW*(*GP*), consists of a series of chemical reactions for cell metabolism, including the anabolic (storing energy) and catabolic (releasing energy) pathways. It is a general pathway that is not cancer-specific. Evidently, the activity of the metabolic pathway in critical-state patients (i.e., those with short-term survival) exhibits a pattern that is distinct from that of non-critical patients (i.e., those with long-term survival) [51]. The metabolic pathway functions based on the activity of biochemical molecules, thus differentially expressed proteins do not appear in the metabolic pathway.

**Table 1:** Risk-active pathways identified *iDRW*(*GP*)

Pathway ID	Pathway name	Total^a^	DE genes	DE proteins	Importance score^b^
hsa01100	Metabolic pathways	1273	220	0	100.00
hsa04115	p53 signaling pathway	68	17	4	28.86
hsa04621	NOD-like receptor signaling pathway	168	30	6	27.44
hsa04218	Cellular senescence	160	30	16	26.07
hsa05203	Viral carcinogenesis	201	50	8	21.07
hsa04066	HIF-1 signaling pathway	100	27	11	20.75
hsa05200	Pathways in cancer	526	131	20	20.05
hsa04714	Thermogenesis	229	37	4	20.01
hsa05120	Epithelial cell signaling in Helicobacter pylori infection	68	17	3	19.19
hsa04926	Relaxin signaling pathway	130	30	9	17.97

^a^Total: the number of genes mapped to the pathway in the KEGG database

^b^Importance score: the importance of a variable measured by out-of-bag (OOB) estimate and it was scaled in 0 to 100

Note that the number of differentially expressed genes (DE genes) and differentially expressed proteins (DE proteins) are also shown (*p*-value of DESeq2 or *t*-test < 0.05)

Table 2 displays the top 10 risk pathways for *iDRW*(*GMP*). Several of these pathways were directly related to the immune system. For example, mitophagy is a selective autophagy process that maintains cell health via the degradation of damaged mitochondria [52–54]. Spliceosomes are a biological unit that facilitates the alternative splicing of pre-mRNA and regulates immune responses [55–57]. Viral inflammation-related pathways were also observed on the list, including systemic lupus erythematosus, toxoplasmosis, and epithelial cell signaling in *Helicobacter pylori* infections. These viral inflammation-related pathways are strongly associated with innate immune responses [58–63] and are commonly accompanied by mitochondrial DNA (mtDNA) mutation [64–70], which is the DNA found in mitochondria that is maternally inherited [53]. Mitochondria are cellular organelles that have a number of roles in a cell, including producing cellular energy, controlling the cell cycle for cell growth and death, biosynthesis, and immunological responses [54]. Abnormal mitochondria function due to mtDNA mutation or depletion could be related to cancer progression. It has been reported that epigenetic modification (such as DNA methylation) controls the expression patterns of mtDNA [53, 71, 72]. Based on our analysis, we conjecture that abnormal methylation in mtDNA is associated with breast cancer.

**Table 2:** Risk-active pathways identified *iDRW*(*GMP*)

Pathway ID	Pathway name	Total^a^	DE genes	DM genes	DE proteins	Importance score^b^
hsa04137	Mitophagy	65	10	12	3	100.00
hsa03040	Spliceosome	134	10	15	1	94.88
hsa05322	Systemic lupus erythematosus	133	24	15	0	90.26
hsa04218	Cellular senescence	160	30	27	16	87.13
hsa04974	Protein digestion and absorption	90	25	17	1	79.23
hsa04622	RIG-I-like receptor signaling pathway	70	10	5	3	79.02
hsa05145	Toxoplasmosis	113	34	11	10	78.72
hsa05120	Epithelial cell signaling in Helicobacter pylori infection	68	17	11	3	72.59
hsa04621	NOD-like receptor signaling pathway	168	30	26	6	69.23
hsa05230	Central carbon metabolism in cancer	65	12	14	8	68.91

^a^Total: the number of genes mapped to the pathway in the KEGG database

^b^Importance score: the importance of a variable measured by out-of-bag (OOB) estimate and it was scaled in 0 to 100

Note that the number of differentially expressed genes (DE genes), differentially methylated genes (DM genes), and differentially expressed proteins (DE proteins) are also shown (*p*-value of DESeq2 or *t*-test < 0.05)

**Fig. 8:**
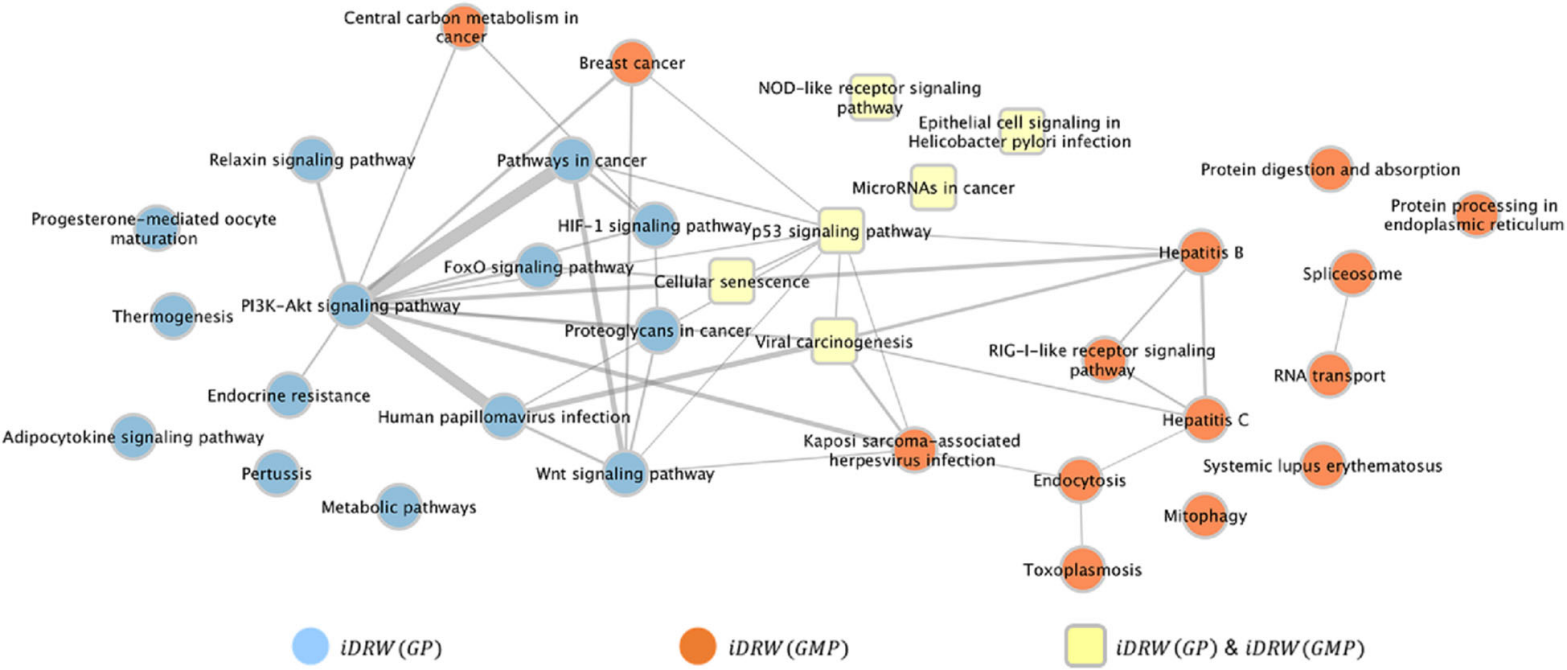
Risk pathway interaction network from *iDRW*(*GP*) and *iDRW*(*GMP*). Risk pathways obtained from *iDRW*(*GP*) and *iDRW*(*GMP*) are shown as blue and orange nodes, respectively, and the common risk pathways in both *iDRW*(*GP*) and *iDRW*(*GMP*) are shown as yellow nodes. Each edge represents pathway-pathway interaction

To investigate the association of the risk pathways identified by *iDRW*(*GP*) and *iDRW*(*GMP*), we created a pathway-pathway interaction network (Fig. 8) with the top 20 risk pathways from each model. The pathway interaction network was constructed using PathwayConnector [73], which is a visualization tool for the direct connection among pathways based on public databases such as KEGG [74] and Reactome [75]. Figure 8 displays the common risk pathways selected by both *iDRW*(*GP*) and *iDRW*(*GMP*). The cellular senescence pathway is a cancer suppressor which regulates cell growth and death by ceasing the division of premalignant or aged cells [47]. We examined the distribution of patients’ ages to determine the relationship between age and long-term/short-term survival. We found that the average age of the long-term survival group was 6 years younger than that for the short-term survival group (*p*-value = 8.233e-05).

Viral carcinogenesis induces tumor progression via oncogenic virus infection [76]. The human papillomavirus (HPV) infection pathway, which is a DNA tumor virus infection, was identified in *iDRW*(*GP*) and has been reported to be closely related to breast cancer, with HPV DNA being found in breast cancer cells [77–79]. It is known that HPV targets tumor suppressor proteins [76]. The infection pathway for another oncovirus, Kaposi’s sarcoma-associated herpesvirus (KSAH or HHV-8), was identified in *iDRW*(*GMP*). It has been reported that KSAH is associated with breast cancer. However, KSAH is an etiologic factor for non-familiar breast cancer [80]. The risk pathways identified by *iDRW*(*GP*) have a relatively strong connection with cancer-related signaling pathways, such as the PI3K-Akt signaling pathway, Pathway in cancer, Proteoglycans in cancer, and the FoxO signaling pathway. The PI3K-Akt signaling pathway is frequently observed in cancer cells and stimulates cell growth and proliferation [81–83], while the FoxO signaling pathway is a tumor suppressor that regulates the genes in cellular physiological processes such as the cell cycle and apoptosis [84–86]. The risk pathways identified by *iDRW*(*GMP*) contain viral infection-related and immune response-related pathways. Though hepatitis B and hepatitis C are not directly related to breast cancer, they cause inflammation of liver tissue, which makes it difficult for cancer patients to receive chemotherapy [87]. This can negatively affect the clinical prognosis for breast cancer patients.

It is known that protein-level data reflects the status of cancer cells better than gene-level data [88]. In this study, we observed that the *iDRW*(*GP*) model mainly reflected the status of cancer cells at the cellular level, while the *iDRW*(*GMP*) model tended to reflect immune response-level information. From our observations, we can infer that the *iDRW*(*GP*) model reflects protein-level information more accurately when compared with *iDRW*(*GMP*). As in [1], it is generally believed that increasing the volume of data for integration leads to further improvements in performance; we hypothesize that, based on the above results, the reason why *iDRW*(*GP*) out-performed *iDRW*(*GMP*) is that the characteristics of DNA methylation hinder the identification of risk pathways which facilitate the prediction of survivability for breast cancer patients that were otherwise discovered by *iDRW*(*GP*). It should be noted that the benefits of different combinations of omics types will depend on the type of clinical problem under examination. Clinical predictions for survival are influenced by both genetic and environmental factors. For survival classification, protein-level information (such as vital signs) is more important than gene-level information (such as innate immune response information).

## Conclusions

In this study, we combined RPPA proteomic data with RNA-Seq and DNA methylation data to successfully derive pathway information based on the iDRW framework. This study found that RPPA data is a rich source of information for survival prediction for breast cancer patients; when RPPA data was employed in the iDRW framework, improved performance was observed and feasible risk pathways extracted. The proposed model successfully identified both well-known and previously undiscovered risk pathways for breast cancer. Systemic analysis was also conducted to obtain better macroscopic insights. Furthermore, network propagation analysis and combinatorial experiments were performed in order to fine-tune our model. We employed network propagation on pathway gene members to overcome the sparsity of RPPA proteins using random walk with restart (RWR). Although the *iDRW*_*prop*_ model did not out-perform *iDRW*, it was robust to the restart probability. The combinatorial experiments assessed the performance of each omics combination, with the (G + P) combination in *iDRW*(*GMP*) producing the highest accuracy overall. Finally, we observed that *iDRW*(*GP*) exhibited the best performance for survival prediction for breast cancer patients and highlighted key differences in the major risk pathways identified using *iDRW*(*GP*) and *iDRW*(*GMP*). These findings highlight that an appropriate combination of omics data is required to properly address the topic under investigation.

## Additional files

Additional file 1: **Supplementary Material 1.** Performance comparison on varying structure of the unified pathways network. (PNG 167 kb)

Additional file 2: **Supplementary Material 2.** Survival curve of *iDRW*(*GM*) and *iDRW*(*GMP*). (a) Survival curve for long-term survival and short-term survival in *iDRW*(*GM*). (b) Survival curve for long-term survival and short-term survival in *iDRW*(*GMP*). (PNG 117 kb)

## Abbreviations

DRW: Directed random walk
G: RNA-Seq profile
iDRW: Integrative directed random walk
iDRW(GM): The model that generates a pathway profile using the RNA-Seq and DNA methylation profiles
iDRW(GMP): The model that generates a pathway profile using the RNA-Seq, DNA methylation, and RPPA profiles
*iDRW*_*prop*_: The model that adopted network propagation for the RPPA weight scores in the proteome network
M: DNA methylation profile
P: RPPA profile
RPPA: Reverse phase protein array
RWR: Random walk with restart
