# Supplementary figures and images for: Topological integration of RPPA proteomic data with multi-omics data for survival prediction in breast cancer via pathway activity inference
Source: BMC Med Genomics. 2019 Jul 11;12(Suppl 5):94. doi: 10.1186/s12920-019-0511-x (PMC6624183; doi:10.1186/s12920-019-0511-x)

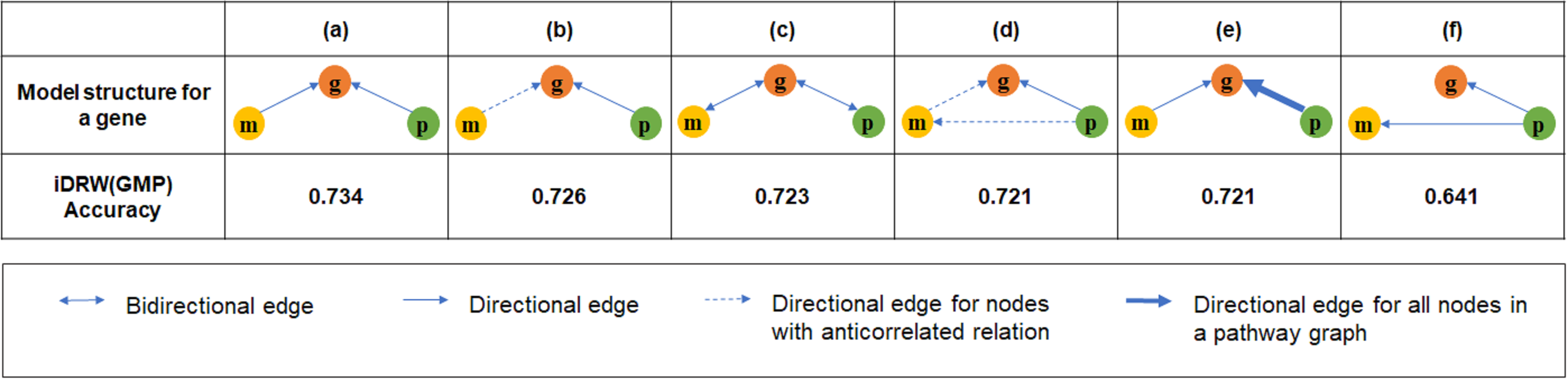

Supplement: Supplementary file 1 — Supplementary Material 1. Performance comparison on varying structure of the unified pathways network. (PNG 167 kb) [file 12920_2019_511_MOESM1_ESM.png]

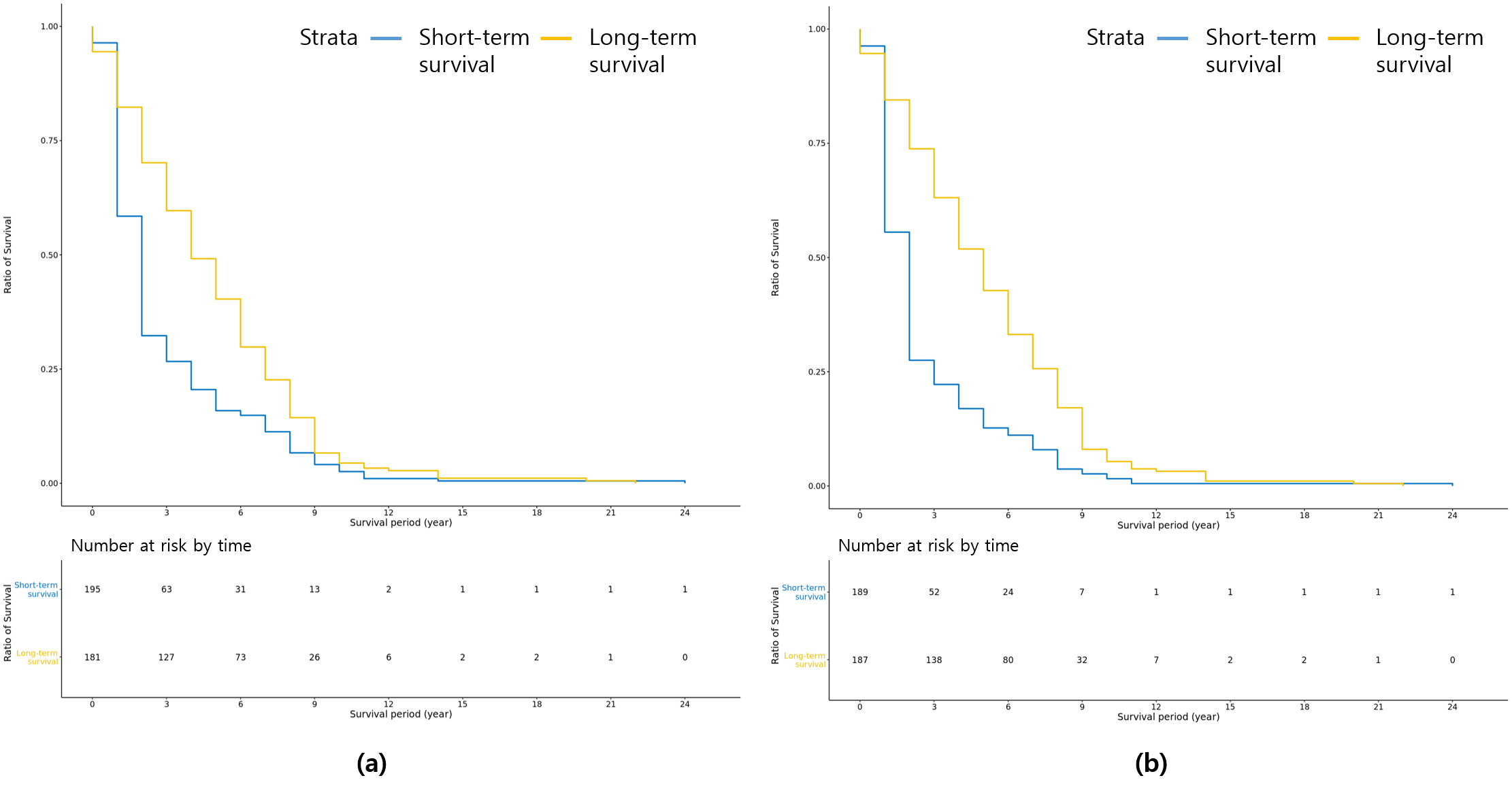

Supplement: Supplementary file 2 — Supplementary Material 2. Survival curve of iDRW(GM) and iDRW(GMP). (a) Survival curve for long-term survival and short-term survival in iDRW(GM). (b) Survival curve for long-term survival and short-term survival in iDRW(GMP). (PNG 117 kb) [file 12920_2019_511_MOESM2_ESM.png]
